# Hypofractionated radiotherapy for lung tumors with online cone beam CT guidance and active breathing control

**DOI:** 10.1186/1748-717X-5-19

**Published:** 2010-02-27

**Authors:** Yali Shen, Hong Zhang, Jin Wang, Renming Zhong, Xiaoqing Jiang, Qinfeng Xu, Xin Wang, Sen Bai, Feng Xu

**Affiliations:** 1 Department of radiation oncology, Cancer centre, West China Hospital, Sichuan University, Chengdu 610041, China; 2 Division of Physics Center, Cancer centre, West China Hospital, Sichuan University, Chengdu 610041, China

## Abstract

**Background:**

To study the set-up errors, PTV margin and toxicity of cone beam CT (CBCT) guided hypofractionated radiotherapy with active breathing control (ABC) for patients with non-small cell lung cancer (NSCLC) or metastatic tumors in lung.

**Methods:**

32 tumors in 20 patients were treated. Based on the location of tumor, dose per fraction given to tumor was divided into three groups: 12 Gy, 8 Gy and 6 Gy. ABC is applied for every patient. During each treatment, patients receive CBCT scan for online set-up correction. The pre- and post-correction setup errors between fractions, the interfractional and intrafractional, set-up errors, PTV margin as well as toxicity are analyzed.

**Results:**

The pre-correction systematic and random errors in the left-right (LR), superior-inferior (SI), anterior-posterior (AP) directions were 3.7 mm and 5.3 mm, 3.1 mm and 2.1 mm, 3.7 mm and 2.8 mm, respectively, while the post-correction residual errors were 0.6 mm and 0.8 mm, 0.8 mm and 0.8 mm, 1.2 mm and 1.3 mm, respectively. There was an obvious intrafractional shift of tumor position. The pre-correction PTV margin was 9.5 mm in LR, 14.1 mm in SI and 8.2 mm in AP direction. After CBCT guided online correction, the PTV margin was markedly reduced in all three directions. The post-correction margins ranged 1.5 to 2.1 mm. The treatment was well tolerated by patients, of whom there were 4 (20%) grade1-2 acute pneumonitis, 3 (15%) grade1 acute esophagitis, 2 (10%) grade1 late pneumonitis and 1 (5%) grade 1 late esophagitis.

**Conclusion:**

The positioning errors for lung SBRT using ABC were significant. Online correction with CBCT image guidance should be applied to reduce setup errors and PTV margin, which may reduce radiotherapy toxicity of tissues when ABC was used.

## Background

Radiotherapy is the alternative treatment for patients with medically inoperable primary non-small-cell lung cancer (NSCLC) [1], and also for patients with slow growing metastatic lung tumors [2] which when managed with high dose localized radiotherapy can prolong patients' symptom-free status.

However, even for inoperable stage I non-small cell lung cancer, the local control rates using standard fractionation schemes (30-76 Gy in 1.8 to 2.0 Gy fractions) have been reported ranging 45-89% [3-5]. Five year actuarial survival of conventional radiotherapy ranged from 6% to 27% [6-9], which was unsatisfactory compared with surgery (with a 5-year survival rate of 60% to 80%) [10]. Dose escalation has been an important issue to improve local tumor control and overall survival [11,12]. However, dose escalation by conventional fractionated radiotherapy has the risk of increasing normal tissue toxicity and prolonging overall treatment time which will encounter the acceleration of tumor cell proliferation.

The dose escalation within a short treatment time and sparing functional lung tissue is potentially addressed by hypofractionated radiotherapy. It has been shown that the use of hypofractionated lung radiotherapy can achieve excellent local control rates as high as 85-95%, with surprisingly minimal acute or late toxicity [13-15]. The hypofractionation radiotherapy technique employs multiple radiation beams to target a tumor with extreme precision, delivering a high dose of radiation, even in a single fraction. Tumors in the thorax regions are subject to setup errors and respiration motion, which can result in inaccurate assessment of organ shape and locations. Conventionally, these uncertainties are accounted for in treatment planning by using large margins based on motion value [16], which can limit dose delivered to tumor.

Special immobilization and verification devices have been developed to reduce setup uncertainties. The use of cone-beam CT (CBCT) has provided 3-dimension information of patient position which could be utilized to guide high precision radiotherapy of the lung tumor. The technique of active breathing control (ABC) has been used to reduce the breathing motion. The use of ABC has been reported to have advantages in protection of lung tissues by reducing respiration motion and lung density [17]. However, little has been reported on the combined use of ABC and CBCT in hypofractionated RT of lung tumor.

Given the availability of onboard cone-beam CT (CBCT) imaging and ABC at our institution, we set out to determine how much using image-guided radiotherapy (IGRT) might affect lung tumor targeting accuracy, target volume margin requirements, and normal tissue doses.

## Methods

### Study population and Characterization

#### Eligibility

Patients with histologically or cytologically confirmed diagnosis of metastatic malignant tumors within the lung or primary NSCLC were eligible for treatment. Patients must have measurable disease and the maximum diameter of tumors is bellow 5 cm. A maximum of 3 lung tumor targets in one patient were allowable. Patients with primary NSCLC either had medically inoperable disease or refused surgery. Patients with metastatic tumors and with life expectancy ≥ 6 months were treated. Patients with a history of prior chest radiotherapy were ineligible. Pretreatment pulmonary function testing was performed, with FEV1 (minimum forced expiration volume at 1 second) ≥ 2.0 L and FEV1/FVC (vital capacity) ≥ 80%. Patients were required to have an ECOG performance status of 2 or less, and not on chemotherapy or hormonal therapy. Informed consent was obtained from all patients before the treatment was initiated.

#### Patient characteristics are shown in Table 1

Between April 2006 and August 2007, 20 patients with inoperable NSCLC or metastatic lung tumors were treated with IGRT at West China Hospital, Sichuan University. Patients comprised 12 males and 8 females aged from 22 to 74 years, with a median age of 54.2 years. Half of the lung tumors were primary NSCLC and the rests were metastatic which came from the head and neck (7), esophagus (2) and breast (1). Of the 10 patients with primary NSCLC, 5 were recurrence after surgical treatment, 3 were ineligible for surgical treatment due to complications and/or advanced age and 2 refused surgery. Ten patients had 1 tumor target, eight patients had 2 targets and the remaining two patients had 3 targets. A total of 32 tumor targets were treated with radiotherapy. The tumor mean size was 23 mm (ranged 13 - 44 mm) on CT scan.

**Table 1:** Patient and tumor characteristics

**Patient**	20
**Tumor**	32
**Age **(yrs)	
Range	22-74
Median	54.2
≥ 60 yrs	8(40%)
**Gender**	
Male 19	12(60%)
Female	8 (40%)
**Resource**	
Primary	10
Metastatic	10
**Histology**	
Squamous cell carcinoma	5 (25%)
Adenocarcinoma	11(55%)
Unclassified	4 (20%)
**Tumor size (mm)**	
Mean	23
Range	13-40
**Tumor location**	
Right lung	20(63%)
Left lung	12(37%)
Upper lobe	12(37%)
**ECOG performance**	
0	14(70%)
1	6 (30%)

### Immobilization and CT simulation

All patients underwent a virtual radiation simulation using a stereotactic body frame (SBF) (Elekta Crawley, UK) for immobilization. A planning CT scan in 3 mm-cuts of the whole thorax was taken, with the patient in the treatment position and using the Elekta ABC device (Elekta, Crawley, UK). To set the threshold of ABC, the patient was told to take a deep breath and the maximum inspiration volume was measured. The breath-hold threshold was set at 3/4 of the maximum inspiration for each patient. Each patient had accepted the training course with ABC for 2-5 times before irradiation. Oxygen with 5-8 L flow rate was connected to the inhale pipe to help patients enhance breath holding time. Patients can release the control switch when he feels uncomfortable. It is required that the respiration motion of tumor with ABC should be < 3 mm as assessed by fluoroscopy before treatment.

### Planning and treatment

Treatment planning was performed using the PrecisePLAN Release 2.1 planning system with considerations made for pulmonary density inhomogeneity. The full area integration dose calculation algorithm was used for dose calculation. Prophylactic nodal irradiation was not performed. Gross tumor volume (GTV) encompassed 1 mm only the radiologically visible tumor as seen by the planning CT with the lung window using a window level of -700 with a width of 1000. Clinical target volume (CTV) was GTV plus a 5 mm margin in all directions. For the planning target volume (PTV), 5 mm security margins in all directions were added to the CTV.

Depending on tumor size and location, different fractionation schemes were applied. There were three groups of different dose per fraction given to the planning target volume (PTV), prescribed to the 80% isodose. In general, radiotherapy with dose per fraction of 12 Gy prescribed was chosen for small targets and for targets with peripheral location. In cases of large tumors, central location and close proximity to critical structures like large vessels and bronchi: with dose per fraction of 6 Gy. Other tumors were given radiotherapy with dose per fraction of 8 Gy. Depend on different single dose we chose different numbers of fractions to make BED (biology effective dose) reach at least 70 Gy. The primary and metastatic lung tumors were not differentially fractionated since in this cohort both primary and metastatic tumors shared similar histopathological types with similar radiosensitivity. The patients received radiotherapy three times per week. The treatment planning ensured that the esophagus, heart and spinal cord received the minimum possible dose, but always less than 50% of the total prescribed tumor dose. According to linear quadratic equation [E/α = nd × [1+d/(σ/β)], BED were calculated and shown in Table 2.

**Table 2:** Radiation therapy fractionations protocols according to Abratt model.

Targets numbers	Dose (Gy) infraction	Number of fractions	BED (Gy)
18	12	4	106
5	8	7	101
3	8	5	72
3	6	10	96
3	6	8	77

* BED: biological effective dose.

### CBCT guidance and adjustment

CBCT was used for verification of tumor position using100 kV, S20 field of view (270 mm), 36.1 mAs, with the kilovoltage source rotating from 260° and ending at 100° for acquisition of 361 frames [18], which was done in one breath-hold.

Before each fraction, a first CBCT was acquired reconstructed and automatically matched to the planning CT. The positional errors of the target in left-right (LR), superior-inferior (SI) and anterior-posterior (AP) axis were calculated with the XVI software. The errors were corrected online through adjustment of treatment couch. The second CBCT was acquired after online correction. If the residual error is less than 2 mm, radiotherapy was delivered immediately. The third CBCT were acquired after radiotherapy to estimate the residual error. The interfractional errors were defined as the offset between the pre-correction CBCT and the planning CT. The intrafraction error was calculated as the difference between the pre- and post-correction position.

### Analysis of positional errors

The inter- and intra-fraction errors are reported as described [19]: for each patient the mean and standard deviation (SD) of all setup errors during treatment were calculated. The group mean error (M) is defined as the average of all individual means. Σ is defined as the variability of the means and calculated as the SD of the individual means. The random uncertainty σ was calculated as the root-mean-square of the individual SD.

### PTV margin reduction and impact on normal tissue dose

As ABC was applied to restrict respiration motion (<3 mm), to simplify analysis, the internal margin (IM) due to respiration motion was not included for margin analysis, only positional uncertainty was accounted for, according to van Herk [19]: M_setup_(PTV margin) = 2.5 Σ + β√σ^2^+σ^2^_p _- βσ_p_, where σ_p _= 6.4 mm for lung, β = 0.84 for SBRT (80% isodose line) [20].

To evaluate the benefit of CBCT-guided online setup correction and ABC device on margin reduction for lung hypofractionated RT, the dose reductions to normal tissues with online correction were simulated in three patients with central, peripheral, and inferior lobe tumor locations, respectively.

### Follow up

Acute toxicity was prospectively assessed for lung, esophagus, and skin using the RTOG acute radiation morbidity scoring criteria every week during treatment. Late lung toxicity was evaluated with a modified scoring system considering only the lung symptoms (Common Toxicity Criteria version 2). Our follow up lasts 16 month. The patients got recheck for chest CT every 3 months. The tumor response was evaluated by a senior radiologist and a radiation oncologist using the RECIST criteria.

### Statistical analysis

F-test was applied for error analysis using SPSS software package.

## Results

A total of 347 CBCT including 150 pre-correction, 130 post-correction and 67 post-treatment scans were acquired. The CBCT images with ABC yielded good contrast of tumor and structures.

At free breathing, the mean (±SD) of diaphragm displacement was 16.0 (±2.7) mm (range 12-24 mm). With the use of ABC, the mean (±SD) diaphragm displacement was 1.7 (±0.5) mm (range 1.0-2.5 mm). Mean value of the breath-hold time was prolonged from 30 seconds to 57 seconds by means of training course and inhaling oxygen in 20 patients. All patients tolerated ABC well for CBCT scan with a duration ≥ 40 s. To make patients more comfortable, the duration of ABC < 20 s is required during radiation delivery. All patients were tested by the respiratory function 3 months after treatment. The result indicated that none of these parameters (FEV1, FEV1/FVC) was affected by IGRT.

### Interfractional errors and intrafracional errors

**Figure 1:**
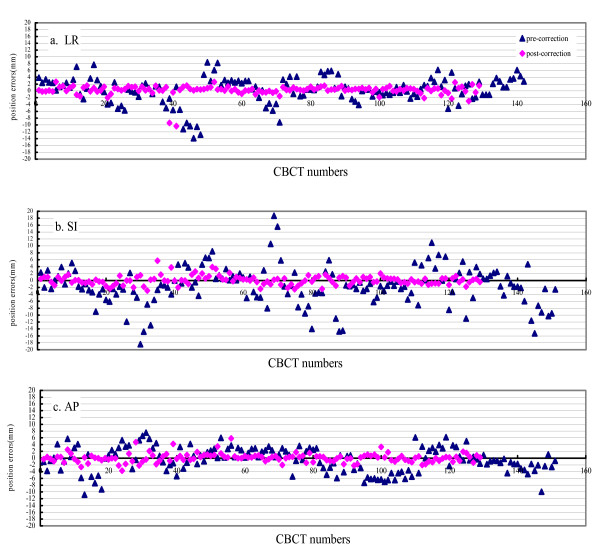
**The tumor positional errors pre- and post-correction, and post-treatment in three dimensions**. The abscissa represents the number of CBCT acquired, and the ordinate represents the errors (in mm), a: LR, b: SI, c: AP.

**Figure 2:**
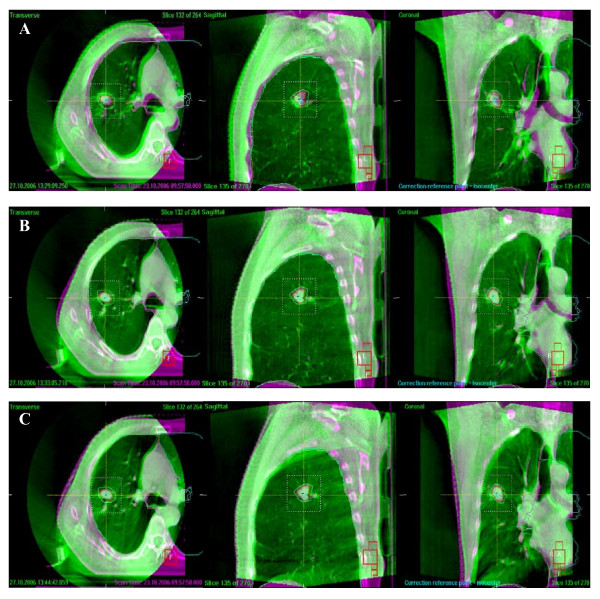
**The ovelapping of targets between simulation CT and CBCT scans of a right upper lung NSCLC**. The pink circle represents the GTV and green circle represents the PTV contours in planning CT. From left to right: transverse, sagital and coronal. A: precorrection, shows shifts of the target position from planning contours. B: Post-correction with online correction, shows satisfactory overlapping of the contours between simulation and CBCT images. C: Post-treatment, the targets in CBCT still overlaps well with the contours in planning CT.

The pre- and post-correction positional errors for patients using ABC in LR, SI and AP directions were shown in Fig. 1 and Fig. 2. It shows that errors in three directions were all decreased with online correction. The percentage of pre-correction errors ≤ 2 mm in all three directions was <30%, while rose to ≥ 90% after correction and remained 60% at the completion of treatment. As shown in Table 3, the pre-correction errors in SI direction were the largest. There was a significant deviation of mean error in the caudal direction, with systematic error of 5.3 mm and random error of 3.7 mm. The precorrection errors were similar in LR and AP directions, with the systematic of 3.1 and 3.7 mm and random error of 2.1 and 2.8 mm for LR and AP, respectively. After correction, the errors were reduced in all three axes. The errors were similar in three axes, with the systematic errors of 0.6 mm in LR and 0.8 mm for both SI and AP direction; and random errors of 0.8 mm, 1.2 mm and 1.3 mm in LR, SI and AP direction, respectively.

**Table 3:** The systematic and random errors and PTV margins in 20 patients pre- and post-correction, and post-treatment.

Setup error	Pre-correction (N = 150)			Post-correction (N = 130)			Post-treatment (N = (67)		
	
	LR	SI	AP	LR	SI	AP	LR	SI	AP
M	-0.3	-1.74	-0.5	0.1	0.1	0.1	-0.4	0.3	0.4
Σ	3.7	5.3	3.1	0.6	0.8	0.8	1.2	1.8	1.2
σ	2.1	3.7	2.8	0.8	1.2	1.3	1.5	1.5	2.8
M_setup_	9.5	14.1	8.2	1.5	2.1	2.1	3.2	4.7	3.5

Abbreviations: N = number of CBCT scans; M = group mean error; Σ = SD of individual means for setup error; σ = root-mean-square of individual SD for setup error; LR = left-to-right; SI = superior-inferior; AP = anterior-posterior; M_setup _= PTV margin for setup. M_setup _(PTV margin) = 2.5 Σ + β√σ^2^+σ^2^_p _- βσ_p_

After treatment, the tumor positional errors increased compared to post-correction (Table 3). The systematic errors were 1.2 mm for both LR and AP axes, and larger (1.8 mm) in SI direction. The random error was larger in AP (2.8 mm) than in LR and SI (both1.5 mm) direction. The mean errors were all below 0.5 mm. The post-treatment residual errors were larger than the post-correction, with increments ≤ 1 mm, but still much smaller than pre-correction errors.

### PTV margin reduction and impact on normal tissue dose

In this study, with ABC device, the respiration motion of tumor was small, only setup uncertainty was included for margin analysis. In Table 3, the pre-correction setup margin was largest (14.1 mm) in SI, intermediate in LR (9.5 mm) and smallest in AP (8.2 mm) directions. The margins decreased markedly after correction to within 3 mm in all directions. After treatment the margins increased to 3.2 - 4.7 mm in three directions.

Table 4 shows the reductions in normal tissue dose volume parameters using online CBCT image guidance for each of the three GTV locations (central, peripheral, and lower lobe). Comparisons are made between different PTV scenarios. In patients using ABC, the reductions of 47-77.3% in lung dose volume endpoints were achieved with CBCT correction. Reductions of 36.3-66.7% in lung dose volume endpoints were achieved when intrafractional setup errors were accounted for. In patients using ABC, reduction in spinal cord doses was highest (55.2-58.5%) for central tumor location and smaller (8.4-17%) for peripheral locations. Comparing to precorrection (PTV1), the dose reductions to normal tissues were greater postcorrection (PTV2) than posttreatment (PTV3). The increment of dose reductions in lung dose volume endpoints ranged 9.1-18.8%, and 3.3-5.9% in spinal cord maximum dose in PTV2 compared to PTV3, suggesting increased normal tissue dose with posttreatment margin due to intrafractional positioning errors.

**Table 4:** Reductions in normal tissue dose volume parameters using online CBCT image guidance and ABC for SBRT of three GTV (16 cc) location

Normal tissue parameter	Dose absolute reduction value			Dose reduction %		
	
	PTV1	PTV2	PTV3	from PTV1 to PTV2	from PTV1 to PTV3	from PTV2 to PTV3
***Mean lung dose(Gy)***						
central location	5.42	2.42	2.91	55.4	46.3	9.1
peripheral location	5.05	2.47	3.22	51.1	36.3	14.8
inferior lobe of lung	11.46	6.07	7.47	47.0	34.8	12.2
***Lung V20 (%)***						
central location	11	2.5	3.7	77.3	66.7	10.6
peripheral location	8	2.5	4	68.8	50.0	18.8
inferior lobe of lung	21	10	12	52.4	42.9	9.5
***Spinal cord Maximum dose(Gy)***						
central location	59.34	24.64	26.58	58.5	55.2	3.3
peripheral location	6.91	5.99	6.32	13.3	8.4	4.9
inferior lobe of lung	22.50	18.67	20.00	17.0	11.1	5.9

**Abbreviations: **PTV1 = precorrection margins with ABC; PTV2 = postcorrection margins with ABC; PTV3 = posttreatment residual margin with ABC+CBCT-correction; V20 = volume of both lungs receiving ≥ 20 Gy.

### Follow up

ll patients were followed up for 6 - 16 months, with a median of 10 months. There was one patient dying of brain metastasis. At 6 months post-treatment, 20 (62.5%) out of 32 targets regressed completely after treatment. 9 (28%) targets shrank more than 30% (PR). 2 (6.3%) targets had SD at 6 months post-treatment, 1 target was not assessed. An overall response rate of 90.6% (29/32) was achieved. The CR was higher in patients with BED ≥ 100 Gy (74%) vs. BED < 100 Gy (33%). The maximum dose of the critical organs was well below the tolerance dose for each organ in the whole group. The maximum value of V20 for the whole group was 21%. The maximum point dose of spinal cord, esophagus and mean lung were 15.7 Gy, 32 Gy and 3.0 Gy respectively. Treatment was well tolerated. Majority of patients did not have treatment related symptoms during and after treatment. 3 (15%) patients had grade 1, 1 (5%) patient had grade 2 acute radiation pulmonary toxicity. 3 (15%) had grade 1 acute esophagitis. All these symptoms alleviated after treatment completion without special treatment. There was no pulmonary or esophageal toxicity of grade 3 or above, no acute skin toxicity and no hemotoxicity during treatment. For late effects, only 2 (10%) patients had grade 1 pulmonary toxicity (imaging change but no symptom), 1 patient (5%) had grade 1 dysphasia. No other late toxicities were observed.

## Discussion

In this preliminary study, we evaluated the feasibility of CBCT guided radiotherapy in combination with ABC to restrict tumor positional error. The role of CBCT guidance in improving treatment accuracy and reduction of target margin requirements for stereotactic lung radiotherapy using ABC procedure was studied.

At initial setup, the tumor positional error was significant even with SBF plus ABC. Our results were similar to literature report that utilized SBF immobilization and portal imaging device to evaluate errors [21] which reported the positioning errors for SBF were 2.3-4.2 mm. Negoro [22] also reported the positioning accuracy ranged 0-8.5 mm, with the mean of 3.2 mm. Our results demonstrated that the initial errors with SBF plus ABC immobilization were greater than those reported recently which also utilized CBCT online guidance and 4D-CT to detect errors in lung tumor immobilized with SBF alone. In their study, the systematic error ranged 2.5 to 3.4 mm and random errors ranged 1.7 to 2.7 mm [16]. This may be partly due to the poor long-term reproducibility of tumor position when repeat CT scans were performed during ABC [23]. It should be mentioned that the value of using the SBF for improving setup accuracy in SBRT is controversial. In a recent study reported by Sonke et al [20], 65 patients with small peripheral lung tumors treated with SBRT without a SBF. In their study the positioning accuracy was evaluated using 4DCT and CBCT imaging, and their results were similar to ours. Although online correction markedly reduced the positional error, the tumor position varied during treatment and might affect the dose distribution in stereotactic radiotherapy. The post-treatment residual systematic errors increased, with the greatest increment of 1 mm in SI direction, and 0.4 mm for both LR and AP direction. The increment of intrafractional random error was 1.5 mm in AP, 0.3 mm in SI and 0.8 mm in LR direction. Only few studies have reported on intrafractional tumor position variation, especially in patients applying ABC. Uematsu et al. [24] used CT scan to measure the intrafraction lung tumor position error and observed that the intrafraction positional variation was small. Guckenberger et al. [25] has utilized CBCT to determine intrafractional error and postulated that 90% of the intrafractional position errors were within 4.8 mm. A recent literature [18] reported that the mean (SD) intrafractional errors of -0.1 mm (1.1 mm), 0.2 mm (1.4 mm) and -0.1 mm (1.5 mm) in LR, SI and AP axes respectively, for thoracic tumors at free breathing, which were smaller than this cohort of patients. This implies that using of ABC might increase the intrafractional patient motion. This might be due to the using of ABC which introduces more procedures and variations [16].

Based on our study, the use of ABC has reduced respiration motion. With ABC, the average diaphragm displacement was significantly reduced from 16.0 mm to 1.7 mm. Hanley et al. [26] has reported that the diaphragm motion was reduced from 26.4 mm to 2.5 mm (0.5-4.9 mm) with ABC procedure. Sarrut [27] reported the lung tumor motion of 0.9-5.9 mm with the use of ABC. Our study also showed high reproducibility of 2 ABC procedures, with a diaphragm movement error of 3 mm. However, there is limitation of using diaphragm position as a surrogate for tumor. This is because that the diaphragm is susceptible to imaging artifacts due to large and rapid motion, and the diaphragm motion may also be influenced by nonrespiratory activity [28].

As ABC was applied to all patients in this study, the tumor respiration motion was small (< 3 mm), the internal target volume (ITV) was considered roughly equal to CTV. Compared to the reliability of tumor motion (average displacement being 1.4 ± 1.0 mm) measured by 4D CT [29], the reliability of tumor motion measured by fluoroscopy is similar. For simplification, the PTV margin calculation only considered setup errors, other error sources such as delineation uncertainty and breathing pattern variation were not accounted for in this study. The pre-correction PTV margin was 9.5 mm in LR, 14.1 mm in SI and 8.2 mm in AP direction. However, it was recommended a uniform PTV margin of 5 mm axial and 10 mm superior-inferior be added for stereotactic lung radiotherapy when image guidance is not used [16]. Our results showed that the margins in three axes all exceeded the recommended margins for a magnitude about 5 mm if CBCT guidance not applied, indicating the necessity of image guidance for accuracy of lung SBRT with ABC. After CBCT guided online correction, the PTV margin was markedly reduced in all three directions. The post-correction margins ranged 1.5 to 2.1 mm which were similar to the literature which utilized CBCT online correction for lung patients [16]. The PTV margins at the completion of treatment were increased as compared to the post-correction margins. The post-treatment margins were 3.2-4.7 mm in three directions. It was suggested by some investigators that at least 5 mm margin should be added for individualized PTV if image guidance and SBF is used [30]. When intra-and interfractional errors were both accounted for, the PTV margin reduction with online correction ranged about 5 to 10 mm in different axes. In our study the online correction resulted in reduction of lung dose volume endpoints of 47-77.3%, and 55.2-58.5% in spinal cord doses for patients using ABC at different tumor locations. It could be inferred from our study that CBCT and online correction can significantly reduce normal tissue doses. As in our study the tumor respiration motion was not evaluated, the benefit of ABC on ITV reduction could not be discussed. It has been indicated that most lung tumors do not exhibit significant motion [31] and there remains inter- breath hold variability in peripheral lung tumor position with the use of ABC inspiration breath hold, which prevents significant PTV margin reduction. However, lung volumes can significantly increase, thereby decreasing the mass of lung within a standard PTV [32]. In addition, ABC may result in a mean relative reduction in lung DVH parameters determining risk of pneumonitis by up to 25% with the potential for safe dose escalation as reported in other study [33]. 4DCT scan has become more popular for SBRT, it has been reported that using mid-ventilation CT scans for treatment planning instead of the conventional free-breathing CT scans, margin reduction is possible, which can reduce the treatment volume up to 50% [34].

Our study found that hypofractionated radiotherapy with BED ranged 72 to 100 Gy could achieve high CR (62.5%), The hypofractionated radiotherapy has radiobiological advantages of counteracting tumor accelerated proliferation. Quite a few researches have been published which showed high local tumor control and surprisingly low toxicities with BED of 100 Gy [35-39]. The response rate in our study seems higher than that reported for NSCLC SBRT, which were assumed to be partly due to the heterogeneity of histopathology in this cohort, since half the cases were metastatic tumors from head and neck, esophagus or breast which were radioresponsive. The small tumor size and small number of cases included in this cohort might be the other contributing factors.

The patients tolerated the treatment well. Only grade 1-2 acute toxicity occurred in 35% of the patients and 15% had grade I late toxicity. Though immobilization device combined with ABC could effectively reduce respiration motion of target, the total margin was not reduced. With the use of online CBCT guided setup correction, PTV margin was substantially reduced, which explained the low toxicity in this patient cohort. Similar results were reported by Fukumoto et al [40] who treated 22 stage I patients with image guided hypofractionated radiotherapy (48-60 Gy in 8 fractions) and found nearly no impairment of pulmonary functions.

In conclusion, for lung cancer hypofractionated radiotherapy using ABC, CBCT guided online correction effectively reduced setup errors and PTV margins. CBCT guidance markedly improved the precision of lung SBRT which might offer a potential dose escalation and effective reduction of normal tissue toxicity.

## Declaration of interests

The authors declare that they have no competing interests.

## Authors' contributions

YS carries out the design of the study and drafting the manuscript; HZ and JW worked on analysis of data; RZ, XJ, QF and XW helped collection of data a; SB and FX contribute equally to the conception of this study and the final approval of the version to be published. All authors read and approved the final manuscript.
